# Particulate matter reduction efficiency analysis of sprinkler system as targeted control measures for construction activity

**DOI:** 10.1016/j.heliyon.2024.e27765

**Published:** 2024-03-19

**Authors:** Young-Bog Ham, Daniel Cheriyan, Hong-Uk Kim, Jae-Goo Han, Young Hyun Kim, P.R. Janani Priyanka, Jae-ho Choi

**Affiliations:** a Heat Pump Research Center, KIMM Institute of Carbon Neutral Energy Machinery, 156 Gajeongbuk-Ro, Yuseong-Gu, Daejeon 34103, South Korea; b Department of Civil Engineering, Dong-A University, S12-401, 550 Bungil 37, Nakdong-Daero, Saha-Gu, Busan, 49315, South Korea; c Energy Systems Research Division, Korea Institute of Machinery & Materials (KIMM), Daejeon 34103, South Korea; d Department of Construction Policy Research, Korea Institute of Civil Engineering and Building Technology, Gyeonggi-Do 10223, South Korea; e ICT Integrated Safety Ocean Smart Cities Engineering Department, Dong-A University, S12-401-1, 550 Bungil 37, Nakdong-Daero, Saha-Gu, Busan, 49315, South Korea

**Keywords:** Particulate matter, Dust control measures, Water sprinkler system, Real-time PM monitoring

## Abstract

Air pollution caused by the construction industry in the form of particulate matter (PM) has increased to an alarming level. The effects on the health of construction workers are found to be hazardous despite the current advancement in construction methods and practices. In particular, the efficiency of existing control measures for reducing PM from various construction activities has not been improved to the desired level. This study investigated the factors that influence the efficiency of a sprinkler system-based control measure when water spraying and dust suppressant solutions are used. The real-time PM exposure was measured during hollow-block cutting activity using Alphanese OPC-N3 sensors in dust chamber. The dust suppressant suppresses dust particles by initially forming a solidified film on the particle surface, and the high cohesion of this film enhances the suppression rate by promoting dust particle coagulation. It was observed that when using a dust suppressant, the PM concentration at 100 bar exceeded concentrations at other pressures, resulting in increased efficacy in reducing PM_10_. Additionally, water spraying at 115 bar was determined to be the optimal control measure for achieving a significant percentage of PM reduction in a shorter period. These findings can be highly beneficial if the water sprinkler system can be modified into a smart mobility-based sprinkler system either ground-based or drone-based at construction sites in improving PM reduction efficiency particularly on high PM emitting activities.

## 1 Introduction

Occupational health hazards have become an increasing concern for governmental organizations and workforce, particularly in the construction industry, owing to the short- and long-term health impairments attributed to the exposure to particulate matter (PM) including hazardous substances [1]. PM in the construction industry comprises a diverse range of particles emitted during various construction activities, posing potential health risks to workers and nearby populations. These particles can include dust, cement, silica, and other contaminants released into the air from processes such as excavation, drilling, cutting, and demolition. The comprehensive review conducted by Ref. [2] sheds light on the sources and exposure levels of PM within construction environments. This underscores the imperative for implementing robust monitoring and control protocols to alleviate potential health risks.

The amount of PM generated from construction sites can vary widely based on several factors – type of construction activities, the scale of the project, the materials used, construction equipment, and local environment conditions. Additionally, regulatory measures and construction practices can influence the amount of PM emissions. However, some studies have attempted to measure PM concentration roughly in specific type of projects [3]. applied a standardized method to characterize the PM emissions arising from three different construction phases – earthworks, superstructure, and finishing – on a multifamily residential building construction site with different aerodynamic diameters (PM2.5, PM10, total suspended particulates (TSP)) [4]. developed an evaluation method to measure the mass concentration of open source PM and the emission factor of PM in different operating areas required for correctly estimating the health risk to construction workers.

Construction workers are continuously exposed to enormous amounts of respirable (aerodynamic diameter of 2.5–10 μm), fine (aerodynamic diameter of 1–2.5 μm), and microparticles (aerodynamic diameter of ≤1 μm). PM exposure, both short- and long-term, is one of the primary health concerns in construction workers [5]. Consequently, workers suffer from various health issues such as asthma, respiratory and cardiovascular diseases, silicosis, chronic obstructive pulmonary disease, lung cancer, and cardiopulmonary diseases [[5], [6], [7], [8], [9]].

Dust pollution from construction activities not only affects the construction workers but also the people residing in neighbourhoods [10,11]. A study conducted by Refs. [12,13,37] on activity-level PM monitoring, considering the dust source and spatio-temporal characteristics of various PM sizes. Their investigation analyzed the fluctuation in PM emissions across diverse construction activities (such as cutting, drilling, mixing, sanding, and plastering) and materials (including hollow block, solid block, wood, and M20 and M25 grade concrete slabs), while also assessing the health risk for workers exposed to varying PM levels. Results show that the highest PM concentration during the drilling activity on the M25 concrete block (4.151 mg/m^3^) was observed and it varies depending on the materials used. These studies suggest that the occupational health hazards in the construction industry require remedial measures particularly for high PM-emitting construction activities [[14], [15], [16]].

There are studies that have validated the effectiveness of PM control measures on construction sites, in connection with the need for such targeted PM control measures [17]. examined local exhaust ventilation (LEV) as a control measure to reduce exposure to respirable quartz during the milling recesses and identified that the average exposure level of respirable quartz was higher than the Dutch limit (equal to 0.075 mg/m^3^). Although the LEV system connected to the recess miller reduced the exposure level, it was still higher than the Dutch limit [12,37]. studied silica dust exposure and its control strategies, discovering levels exceeding the 0.05 mg/m^3^ standard set by the American Conference of Governmental Industrial Hygienists (ACGIH).

[18] developed a truck-mounted water spray system to control the dust during material movement in the mining industry. The authors assessed the optimum pressure of the system for efficient dust mitigation (water consumption rate of 0.011–0.013 m^3^/min at 2.8 kg/cm^2^) [19]. utilized watering as a countermeasure for earthmoving activities. They found that a 2-h water spraying session led to a 75% reduction in dust [20]. monitored the concentration reduction of PM_2.5_ and TSP, when artificial water sprinkling and water spray systems were used; these levels were reduced by 72.01% and 40.16% for PM_2.5_ and TSP, respectively [21]. studied the efficiency of a single targeted control measure (DustBubbles) in reducing the PM generated during the drilling of concrete and obtained an efficiency of 63%. However, those studies did not consider the propagational characteristics of PM and the variation in PM reduction with the applied water pressure.

In terms of applying multiple control measures [22], stated that a 70% reduction in dust exposure can be achieved using a combination of two control measures, such as LEV and dust reduction by water [14]. recommended the use of multiple PM control measures, such as water spraying systems, fans, and earplugs. These studies suggest that the efficiency of individual control measures is necessary for implementing targeted PM control measures for high-PM-emitting activities and thus, reducing the cost of implementing multiple control measures [23]. stated that water spraying has fewer cost savings compared to the use of dust palliatives at mining sites. Moreover, labor and fuel expenses for the water trucks [24], and an excessive amount of water use at the mining sites [25] are major cost concerns for construction managers.

[26] emphasized the need for targeted dust control measures to reduce PM levels by identifying the source of dust generation, while also noting that costs are a significant factor to be considered during the implementation of such measures [27]. assessed that improper application of water for dust suppression led to water wastage, reflecting a lack of knowledge on PM monitoring and targeted control [12,28,37]. suggested the implementation of targeted PM control measures for activities with higher PM emissions (cutting and mixing) by considering the appropriate location of the PM control measures (water sprinkling and LEV).

Therefore, it is essential to identify the optimum pressure for a water spraying system at which the highest PM reduction can be obtained. Additionally, proper implementation of the control measures to maximize efficiency without causing additional costs is highly beneficial for construction managers, leading to a reduction in PM exposure and minimizing potential health impacts. Therefore, this study focuses on improving the efficiency of the sprinkler system used as a PM control measure by considering execution factors (spraying pressure, location of the sprinkler system, and spraying liquid). The authors used a real-time, location-based, and experimental PM monitoring setup to measure PM concentrations produced from a hollow-block cutting activity.

## 2 Methodology

**Fig. 1:**
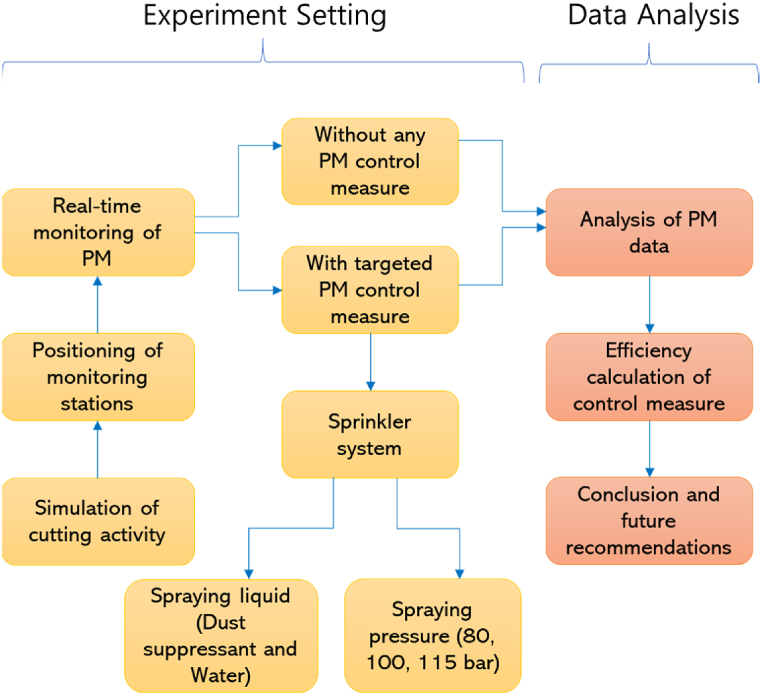
Methodology of the study.

The authors experimentally investigated the efficiency of a targeted PM control measure (sprinkler system); the methodology is shown in Fig. 1. The total cost of the sprinkler system was about 1000 USD, including the cost of nozzles, metal parts, a pressure regulator, a voltage controller, and other costs such as tank, power supply parts, and spraying liquid. The liquid consumption rate and the sprinkler system cost were found to be economical compared to previous research [18].

Initially, cutting activity was performed on a hollow block of dimensions 40 cm × 20 cm × 10 cm to generate PM. The hollow block cutting activity is the most frequent architectural construction work, producing a sufficient amount of different types of PM in a relatively short period of time. PM concentration monitoring stations were selected by considering the spatio-temporal characteristics of PM particles [12,37]. The real-time PM exposure was measured during the experiments with and without targeted PM control measures using a real-time PM sensor (an Alphanese OPC-N3 sensor). As shown in Fig. 1, two variables were considered for assessing the efficiency of the sprinkler system in this study: type of spraying liquid (dust suppressant and water) and spraying pressure (80 bar, 100 bar, and 115 bar). The efficiency of the sprinkler system was calculated by analyzing PM data obtained from real-time sensors.

### 2.1 Materials and devices

The hollow-block cutting activity was conducted in the laboratory of Dong-A university. A D25133K model cutting machine was used to cut to a depth of 4 mm. The PM concentration was measured by real-time Alphanese OPC-N3 sensors [ [12,29,30,37]]. The specifications of the sensor are shown in Table 1. The sensor measures PM_10_, PM_2.5_, and PM_1_ particles simultaneously, and is more sensitive to low PM concentrations [30]. The cutting activity generated more PM_10_ particles compared to the finer particles (PM_1,_ and PM_2.5_). Therefore, we considered only PM_10_ concentration for assessing the efficiency of the control measure in this study as the reduction in PM_10_ particles would suppress the higher proportion of the generated dust.

**Table 1:** Specifications of Alphanese OPC-N3 sensor.

Technical data	Alphanese OPC-N3
Particle range (μm)	0.38–17
Particle size (μm/m^3^)	PM_10_, PM_2.5_, and PM_1_
Sampling interval (s)	1–30
Maximum particle count rate (particles/s)	10000
Data storage	micro-SD (.CSV format)
Temperature range (°C)	−10 to 50
Humidity range (% RH)	0–95
Weight (g)	<105
Usage	Static/movable

A sprinkler system was used for dust suppression, and the pressure of the liquid particles was regulated using a pressure controller connected to the spraying system. The water and dust suppressant were filled into the sprinkler system. The water used in this study was filtered water, and the dust suppressant was developed from Chitosan, a solution obtained from seashells [[31], [32], [33]]. This solution was diluted with water in a ratio of 1000:1 in this study [34]. The dust suppressant liquid has been proven to be eco-friendly, nontoxic, and nonflammable [31,32]. The dust suppressant suppresses dust particles through the solidification process by initially forming a solidified film on the dust particle surface. The high cohesion of the solidified film increases the suppression rate by promoting the coagulation of the dust particles [32].

### 2.2 Experimental setup and execution

The experimental setup was adopted from the research article [12,37]. A dust chamber of dimensions 4.0 m × 4.0 m was built inside the professional construction management service laboratory in Dong-A University in Korea to monitor the PM concentrations. The dust chamber was built to minimize the effect of temperature, humidity, and wind speed on the reliability of real-time sensor data. The execution was based on the double replicate of single-level factorial experimental design [ [12,35,37]]. We selected one level for both the horizontal and vertical distances of 1 m, based on the distributional properties of PM and height at which people may breathe [12,37].

**Fig. 2:**
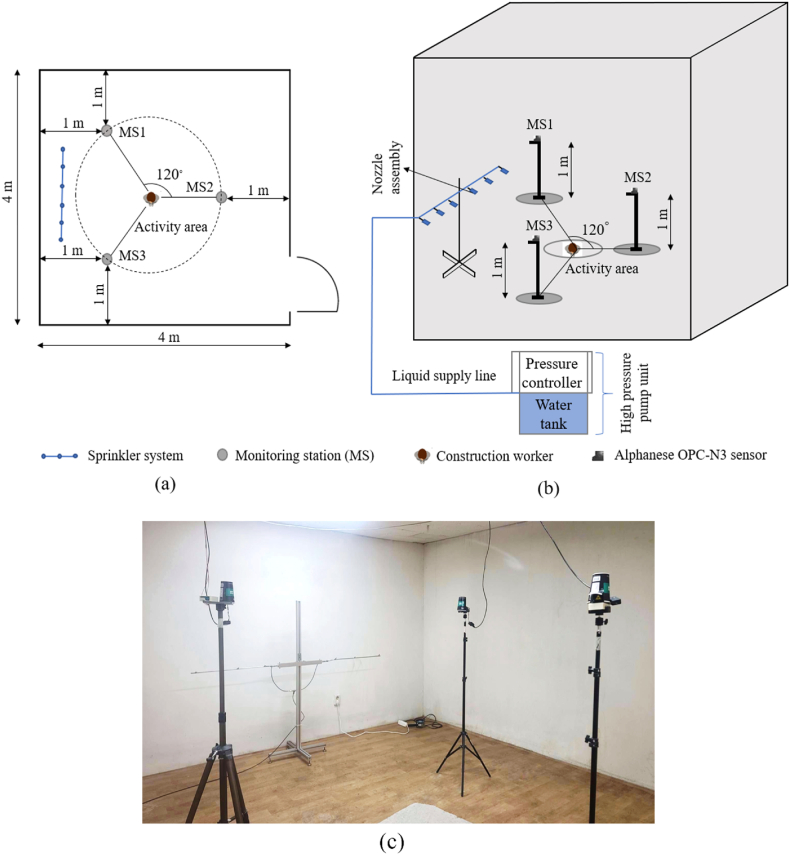
(a) positioning of the monitoring stations, (b) schematic representation of the dust chamber, and (c) experimental setup.

Three monitoring stations were positioned at 1 m height to absorb the high concentration of PM exposure and were placed inside the dust chamber at 120° with respect to each other and 1 m away from the dust chamber walls to reduce the deflection of particles (refer to Fig. 2a). The sprinkler system was installed inside the dust chamber at a height of 1 m such that the liquid was sprayed in the range of breathing height (0.8 m–1.5 m), using a high-pressure pump unit placed outside the chamber (Fig. 2b and c). The pressure of the sprinkler system was regulated manually using the pressure controller. Each monitoring station was equipped with one Alphanese OPC-N3 sensor. The sensors were calibrated with the benchmark dust sensor (Kanomax monitor model 3443) to reduce uncertainty in the measured data [30]. The Alphanese OPC-N3 sensors exhibited a correlation value of more than 0.80 with the Kanomax monitor. In addition, the correlation among the three Alphanese OPC-N3 sensors was tested and a correlation value greater than 0.90 was obtained.

The PM concentration was observed at a single level at each monitoring station to study the variation in the PM concentration during the experiment. Each activity was repeated twice to confirm the accuracy of the findings. Prior to each experiment, dust and humidity levels were monitored inside the laboratory and in the dust chamber. The experiment was conducted only if the PM level was lower than the reference level; else, the experiment was conducted on the next day. After each experiment, exhaust ventilation and vacuum were used to reduce the PM level inside the dust chamber [12,37].

The humidity level inside the laboratory was above 80% before the experiments. The deflection of particles in a normal environment would be higher. Initially, we used adhesive sheets on the floor and walls of the chamber and dehumidifiers to reduce the humidity. Water was sprayed on the adhesive sheets to minimize the deflection of dust particles. The adhesive sheets absorbed the sprayed water and the moisture content inside the chamber, increasing the humidity level. As a result, the accuracy of the recorded PM data was reduced (the Alphanese OPC-N3 sensor showed errors in the collected PM data at a humidity above 75%) [36]. Moreover, after the sprinkling activity, the humidity level was above 85% because of the absorption of sprayed liquid particles by the adhesive sheets. Therefore, we removed the adhesive sheets; water was sprayed on the wooden surfaces of walls and floor of the chamber to reduce the deflection of particles and the humidity level was set at 50% before starting the experiments.

The real-time PM_10_ concentration was monitored during hollow-block cutting without any control measures, with spraying water and dust suppressant solutions as control measures. The experiments were conducted at different pressures (80 bar, 100 bar, and 115 bar) to identify the optimal pressure for efficient reduction in PM in a short time period. Each activity was conducted for 2 min and 30 s for the spraying system as the control measure, the sprinkler system was operated at a particular pressure for 1 min and 30 s. The consumption rate of the liquid during the sprinkling activity was 0.0011 m^3^/min at 115 bar pressure, 0.00077 m^3^/min at 100 bar pressure, and 0.00033 m^3^/min at 80 bar pressure. The reduction in PM_10_ concentration was then monitored for more than 1.5 h from the start of the activity.

## 3 Results and discussion

### 3.1 Spraying water and dust suppressant at 100-bar pressure

**Fig. 3:**
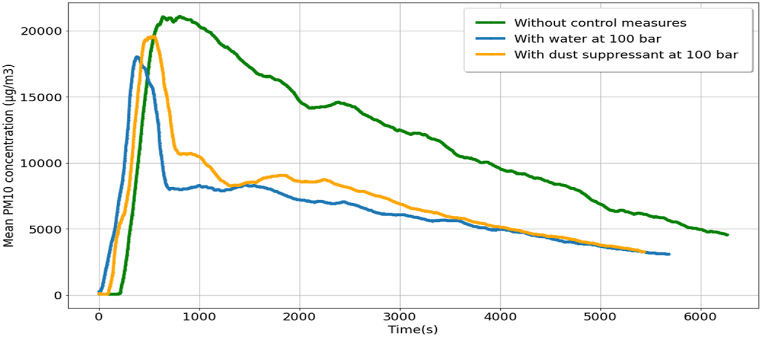
Mean PM10 concentration for cutting activity without control measures and with control measures of water and dust suppressant at 100 bar pressures.

The mean PM_10_ concentrations obtained for cutting without any control measure, water spraying as a control measure, and dust suppressant spraying as a control measure are illustrated in Fig. 3. The trends obtained in all three cases are similar. At the start of the cutting, the PM concentration increases even after the water or dust suppressant sprinkler system was used. The maximum PM_10_ concentrations obtained for cutting with spraying water and dust suppressant at 100 bar were 18033.48 μg/m^3^ and 19578.64 μg/m^3^, respectively. For the case of cutting without control measure, the maximum PM_10_ concentration was 21081.56 μg/m^3^. The PM_10_ reduction was slow for the activity without control measure. The lowest PM concentration after reduction was 4550.71 μg/m^3^. The trends of PM_10_ reductions for water and dust suppressants were found to be similar; PM_10_ reduction was faster at the start compared with the reduction without control measure. Initially, the concentration decreased abruptly, then decreased at slower rates; the PM_10_ concentration at the end of monitoring was 3266.70 μg/m^3^ and 3085.11 μg/m^3^ for cutting with spraying the dust suppressant and water, respectively (refer to Fig. 3).

Table 2 lists the details of cutting activity without control measures. The activity started at 11:51:30 and ended at 11:54:00, and the PM monitoring was performed until 13:35:30. To reach a 30% reduction in PM_10_ concentration, the time required was 50 min and 48 s after the cutting activity. Reduction rates equal to 50% and 70% were attained after 1 h 17 min 10 s and after 1 h 22 min 19 s, respectively, from the end time of the activity. The maximum percentage PM reduction was 78% after 1 h 40 min 14 s. These results indicate that more time was required to reach a reduction in PM concentration greater than 70% for activities that did not involve any control measure.

**Table 2:** Details of cutting activity without control measures.

Event	Time (h:min:s)	The amount of time required for PM reduction after activity (h:min:s)
Start of activity	11:51:30	–
End of activity	11:54:00	
End of PM monitoring	13:35:30	
30% PM reduction	12:44:48	00:50:48
50% PM reduction	13:11:10	01:17:10
70% PM reduction	13:16:19	01:22:19
78% PM reduction	13:34:14	01:40:14

The details of the experiment with water and dust suppressant spraying as a control measure at 100 bar pressure are listed in Table 3. A 30% reduction was attained at 5 min 24 s after the start of the sprinkling of water. Compared with the dust suppressant, a 30% reduction required 2 min 55 s more than that by the activity with water as a control measure. The maximum PM reduction was 83%, according to which the dust suppressant required a smaller time period (2 min 37 s) than that required by water (see Table 3). These results highlight the fact that the dust suppressant at 100-bar pressure achieved efficiencies >70% faster when compared with that by water at 100-bar pressure. However, water is more efficient compared with dust suppressants for 30% and 50% reductions in PM.

**Table 3:** Details of cutting activity with control measures (water and dust suppressant sprayed at 100 bar pressure).

Event	Time (h:min:s)		Time required for PM reduction after sprinkling (h:min:s)	
	With water at 100 bar	With dust suppressant at 100 bar	With water at 100 bar	With dust suppressant at 100 bar
Start of activity	14:15:30	14:59:00	–	–
Start of sprinkling	14:18:00	15:01:30		
End of sprinkling	14:19:30	15:03:00		
End of PM monitoring	15:50:00	16:33:00		
30% PM reduction	14:24:54	15:11:19	00:05:24	00:08:19
50% PM reduction	14:25:48	15:17:28	00:06:18	00:14:28
70% PM reduction	15:15:43	15:57:24	00:56:13	00:54:24
78% PM reduction	15:32:21	16:15:46	01:12:51	01:12:46
83% PM reduction	15:48:04	16:28:57	01:28:34	01:25:57

### 3.2 Optimum pressure for dust suppressant spraying

**Fig. 4:**
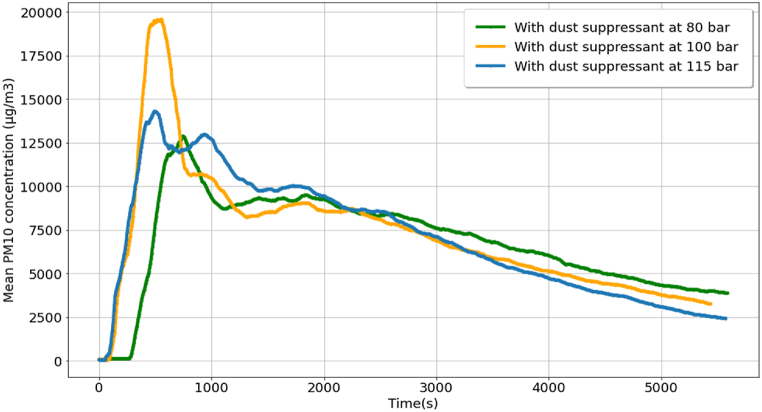
Mean PM10 concentration for cutting activity with dust suppressant (sprayed at 80 bar, 100 bar, and 115 bar pressures) as the control measure.

The experiment was continued by spraying dust suppressant as a control measure at pressures of 80 bar, 115 bar, and 100 bar, and the results are shown in Fig. 4. The maximum mean PM_10_ concentrations after spraying the dust suppressant were 12887.53 μg/m^3^ and 14298.46 μg/m^3^ at 80 bar and 115 bar, respectively. The maximum mean PM_10_ concentration observed for the dust suppressant sprayed at 100 bar was the highest (19578.64 μg/m^3^). The PM levels dropped quickly from their maximum values and in each case, the reduction slowed down, but persisted at a smaller rate. However, at the end of the PM monitoring, the PM_10_ concentrations were less than 4000 μg/m^3^ (3892.15 μg/m^3^, 3266.70 μg/m^3^, and 2427.99 μg/m^3^ for dust suppressants at 80 bar, 100 bar, and 115 bar, respectively).

Table 4 shows details of cutting activities with control measures as the dust suppressant was sprayed at 80 bar and 115 bar, respectively. The dust suppressant at 80 bar started at 15:31:00, and the sprinkling of the dust suppressant ended at 15:35:00. The PM monitoring was completed by 17:04:00. Reductions of 30% and 50% in PM were observed after 13 min 5 s and 56 min 29 s from the time of completion of the sprinkling activity, respectively. The maximum percentage of reduction in PM recorded was 70% (after 1 h 28 min 15 s after the dust suppressant sprinkling). In the case of dust suppressant at 115 bar, the maximum reduction in PM was 83%, similar to the result for dust suppressant at 100 bar (refer to Table 3).

**Table 4:** Details of cutting activities with dust suppressant spraying at 80 bar and 115 bar pressures as the control measure.

Event	Time (h:min:s)		Time required for PM reduction after sprinkling (h:min:s)	
	With dust suppressant at 80 bar	With dust suppressant at 115 bar	With dust suppressant at 80 bar	With dust suppressant at 115 bar
Start of activity	15:31:00	15:21:30	–	–
Start of sprinkling	15:33:30	15:24:00		
End of sprinkling	15:35:00	15:25:30		
End of PM monitoring	17:04:00	16:54:00		
30% PM reduction	15:48:05	15:43:45	00:13:05	00:18:15
50% PM reduction	16:31:29	16:10:19	00:56:29	00:44:49
70% PM reduction	17:03:15	16:31:16	01:28:15	01:05:46
78% PM reduction	–	16:43:16	–	01:17:46
83% PM reduction	–	16:52:48	–	01:27:18

The assessment of PM reduction efficacy for dust suppressants at pressure levels of 80 bar, 100 bar, and 115 bar revealed that the optimum pressure was 100 bar. At 80 bar, the dust suppressant achieved the lowest maximum PM reduction, registering at 70%. Conversely, the applications at 100 bar and 115 bar demonstrated mean PM10 concentration reductions of up to 83%. Notably, the dust suppressant at 100 bar exhibited an 83% PM reduction rate, outpacing the performance of the suppressant at 115 bar (refer to Table 3, Table 4). This observation underscores a trend where increasing dust suppressant pressure correlates with a diminishing efficiency of PM reduction. Furthermore, efficiencies were observed to decrease when the dust suppressant pressure fell below 100 bar.

**Fig. 5:**
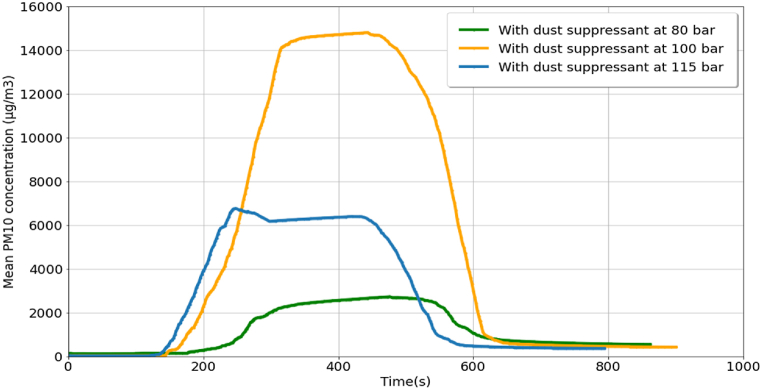
Mean PM10 concentration from experiments with dust suppressant sprayed at 80 bar, 100 bar, and 115 bar pressures.

To validate this finding, experiments were conducted using the sprinkler system without performing cutting. The sensors recorded the concentration of sprayed particles as PM_10_ dust particles as shown in Fig. 5. A higher amount of PM_10_ particles was emitted from dust suppressant spraying at 100 bar, whereas a lower amount of PM_10_ particles was emitted at 80 bar. The highest concentrations recorded were 2728.15 μg/m^3^, 14798.40 μg/m^3^, and 6759.92 μg/m^3^ for experiments with dust suppressant sprinkling at 80 bar, 100 bar, and 115 bar, respectively. These results indicate that as the concentration of the sprayed dust suppressant particles was higher at 100 bar, more PM_10_ dust particles were removed at this pressure compared with that at the other two pressures. The sizes of dust suppressant particles at 100 bar was substantially similar to that of PM_10_ dust particles, thus indicating that PM_10_ dust particles decreased at faster rate at 100 bar. When the dust suppressant pressure was higher or lower than 100 bar, the particle size of the dust suppressant decreased compared with the size of PM_10_ dust particles, and the PM reduction efficiency reduced [18].

### 3.3 Optimum pressure for water spraying

Supplementary material (S1) illustrates the mean PM10 concentration recorded for water spraying as a control measure at 80 bar, 100 bar, and 115 bar. After the sprinkling of water, the mean PM_10_ concentration increased and reached a maximum value. The maximum concentrations attained for water spraying at 80 bar, 100 bar, and 115 bar were 16424.03 μg/m^3^, 18033.48 μg/m^3^, and 30148.26 μg/m^3^, respectively. It is evident that the experiment with water spraying at 115 bar resulted in a higher maximum PM_10_ concentration. After reaching the peak PM concentration, PM levels initially reduced at a faster rate and then slowly attained minimum PM concentrations equal to 2606.83 μg/m^3^, 3085.11 μg/m^3^, and 1646.41 μg/m^3^ for water spraying at 80 bar, 100 bar, and 115 bar, respectively.

Table 5 illustrates the details of cutting activity with water spraying as a control measure at 80 bar and 115 bar. The maximum percentage of reduction in PM was 83% for water spraying and 70% for dust suppressant spraying at 80 bar (refer Table 4, Table 5). Additionally, a 70% reduction for water at 80 bar required 25 min 40 s less than dust suppressant at the same pressure. This indicates that water spraying is more efficient at 80 bar. Moreover, 83% PM reduction for water at 80 bar was attained after 1 h 31 min, and 36 s from the completion of sprinkling activity; the time required was 3 min and 2 s greater than that for water at 100 bar (refer Table 3, Table 5). This clearly indicates that the water spraying at 115 bar is the optimum option as a control measure for high percentage of PM reduction in a shorter period of time.

**Table 5:** Details of cutting activity with water spraying at 80 bar and 115 bar pressures as the control measure.

Event	Time (h:min:s)		Time taken for PM reduction after sprinkling (h:min:s)	
	With water at 80 bar	With water at 115 bar	With water at 80 bar	With water at 115 bar
Start of activity	15:45:00	15:19:00	–	–
Start of sprinkling	15:47:30	15:21:30		
End of sprinkling	15:49:00	15:23:00		
End of PM monitoring	17:21:00	16:50:00		
30% PM reduction	15:59:08	15:30:41	00:10:08	00:07:41
50% PM reduction	16:30:34	15:31:37	00:41:34	00:08:37
70% PM reduction	16:51:35	15:50:49	01:02:35	00:27:49
78% PM reduction	17:04:24	16:01:47	01:15:24	00:38:47
83% PM reduction	17:20:36	16:10:31	01:31:36	00:47:31
95% PM reduction	–	16:49:45	–	01:26:45

The results showed that 1 min 30 s of water sprinkling reduced the PM_10_ exposure by 95%, compared to a 75% reduction in PM_10_ after 2 h of water spraying in the study by Ref. [19]. Moreover, in contrast to the suggestions of [14,22], in cases wherein combinations of control measures are not implemented, more than 90% reduction in PM can be achieved using a single control measure at appropriate conditions. In this study, the higher percentage of PM reduction was attained by considering the propagation characteristics of PM particles and execution factors such as the sprinkling pressure and positioning in the case of a sprinkler system as a targeted control measure.

Similar to dust suppressant spraying, the experiments were conducted with water spraying at 80 bar, 100 bar, and 115 bar without performing the activity (refer S2). Water spraying at 115 bar generated more water particles similar to the size of PM_10_ particles (that is, 32686.74 μg/m^3^). The highest concentration of water particles at 80 bar and 100 bar were 17059.08 μg/m^3^ and 27162.17 μg/m^3^, respectively. This indicates that as the pressure of water spraying increases, the size of water particles becomes relatively similar to PM_10_ particles. Thus, these water particles would carry the PM_10_ dust particles more easily and settle down faster [18].

### 3.4 Efficiency calculation

The efficiency of spraying water and dust suppressant solution as a dust control measure at 80 bar, 100 bar, and 115 bar was calculated with respect to the time taken for 30%, 50%, 70%, and 78% PM reductions in case of activity executed without any control measure. The determined efficiency values are listed in S3 and plotted in S4. Considering the efficiencies achieved for PM_10_ reductions, the determined values were the lowest in the case of dust suppressant spraying at 80 bar. However, for a 30% reduction, the efficiency was lowest for dust suppressant sprinkling at 115 bar. These results indicate that water spraying is a more effective targeted dust control measure at a higher pressure, such as 115 bar, and the efficiency of PM reduction reduces with a decrease in water-particle pressure. Similarly, dust suppressant spraying at 100 bar yielded efficient results compared with the other two pressures. The efficiency of PM_10_ reduction was observed to decrease at a higher or lower pressure than 100 bar in the case of dust suppressant. Dust suppressant sprinkling at 100 bar was slightly more efficient than water sprinkling at 100 bar for PM_10_ reductions of 70% and 78%. However, the efficiency was the highest for water compared with the dust suppressant in all the other cases investigated in this study (refer to S3 and S4).

## 4 Conclusions

Air pollution in the construction industry and methods for its control have gained remarkable attention in the past few decades. Several studies have attempted to reduce PM exposure by adopting various control strategies. However, the improper application and positioning of a countermeasure will not efficiently reduce PM. In this study, spraying water and a dust suppressant solution were employed as targeted PM control measures to reduce the PM concentration arising out of a hollow-block cutting activity. The sprinkler system was activated for 1 min 30 s at three different pressures (80 bar, 100 bar, and 115 bar). The concentration of PM_10_ particles was monitored using an Alphanese OPC-N3 real-time sensor in the lab setting explained in the methodology section.

Throughout this investigation, several crucial experimental findings have come to light. Firstly, the application of water sprinkling proved to be effective in achieving a 50% reduction in particulate matter (PM) compared to the utilization of dust suppressant. However, both methods exhibited similar effects once PM concentration reduction exceeded this threshold. Secondly, when employing dust suppressant, the PM concentration at 100 bar surpassed that at other pressures, leading to a higher efficacy in reducing PM_10_. Thirdly, the optimal control measure for achieving a substantial percentage of PM reduction in a shorter duration was identified as water spraying at 115 bar. Lastly, water spraying emerged as a more effective targeted dust control measure at higher pressures, such as 115 bar, while the efficiency of PM reduction diminished with a decrease in water-particle pressure.

In particular, this study conducted experiments without cutting operations under pressures of 80 bar, 100 bar, and 115 bar for two methods: water spraying and dust suppressant solutions. For the former, PM_10_ emissions were highest at 115 bar, while for the latter, they peaked at 100 bar. This can be interpreted in conjunction with the fact that PM_10_ reduction efficiency was highest at 115 bar and 100 bar, respectively, for each method. In line with [18], they explained that a heightened ratio of sprayed particle size to dust particle size is associated with a significant reduction in PM_10_ concentration, and an increase in the quantity of sprayed particles relative to dust particles enhances the overall efficacy of PM reduction. From this perspective, the experimental results of this study align precisely with [18] assertion for water spraying solutions, while for dust suppressant solutions, the results indicate the presence of an optimal pressure.

These insights contribute valuable knowledge to the realm of dust control measures in construction activities. In addition, this study can be regarded as highly meticulous in measuring the PM reduction effects of two methods, water spraying and dust suppressant solutions, more scientifically and accurately at the experimental scale, while minimizing external meteorological influences. However, to enhance the objectivity of the study results, it is imperative to augment the number of experiments conducted for each case, ensuring a statistically significant level of repetition. The primary impact of the dust suppressant solution utilized in this investigation is the formation of a biofilm on fine dust surfaces, mitigating their re-dispersion due to factors like wind. Therefore, for a comprehensive analysis of the PM reduction effect, additional research endeavours are warranted, considering the efficacy in preventing the re-scattering of PM at construction sites. At last, these findings could offer significant advantages if the water sprinkler system is adapted into an intelligent, mobility-centric sprinkler system, whether ground-based or drone-based, for construction sites. This adaptation has the potential to enhance PM reduction efficiency, especially during high PM-emitting activities as a targeted control PM measure.

## Data availability statement

Data will be made available on request.

## CRediT authorship contribution statement

**Young-Bog Ham:** Funding acquisition, Data curation. **Daniel Cheriyan:** Investigation, Formal analysis, Data curation, Conceptualization. **Hong-Uk Kim:** Project administration, Data curation. **Jae-Goo Han:** Resources, Investigation. **Young Hyun Kim:** Resources, Investigation, Conceptualization. **Jae-ho Choi:** Writing – review & editing, Supervision, Methodology, Funding acquisition, Conceptualization.

## Declaration of competing interest

The authors declare the following financial interests/personal relationships which may be considered as potential competing interests: Jae-ho Choi reports financial support was provided by 10.13039/501100007694Korea Agency for Infrastructure Technology Advancement (10.13039/501100007694KAIA). Jae-ho Choi reports financial support was provided by 10.13039/501100003725National Research Foundation of Korea (10.13039/501100003725NRF). If there are other authors, they declare that they have no known competing financial interests or personal relationships that could have appeared to influence the work reported in this paper.
