# Supplementary material for: Particulate matter reduction efficiency analysis of sprinkler system as targeted control measures for construction activity
Source: Heliyon. 2024 Mar 19;10(7):e27765. doi: 10.1016/j.heliyon.2024.e27765 (PMC10979197; doi:10.1016/j.heliyon.2024.e27765)
Supplement: Multimedia component 1 [file mmc1.docx]

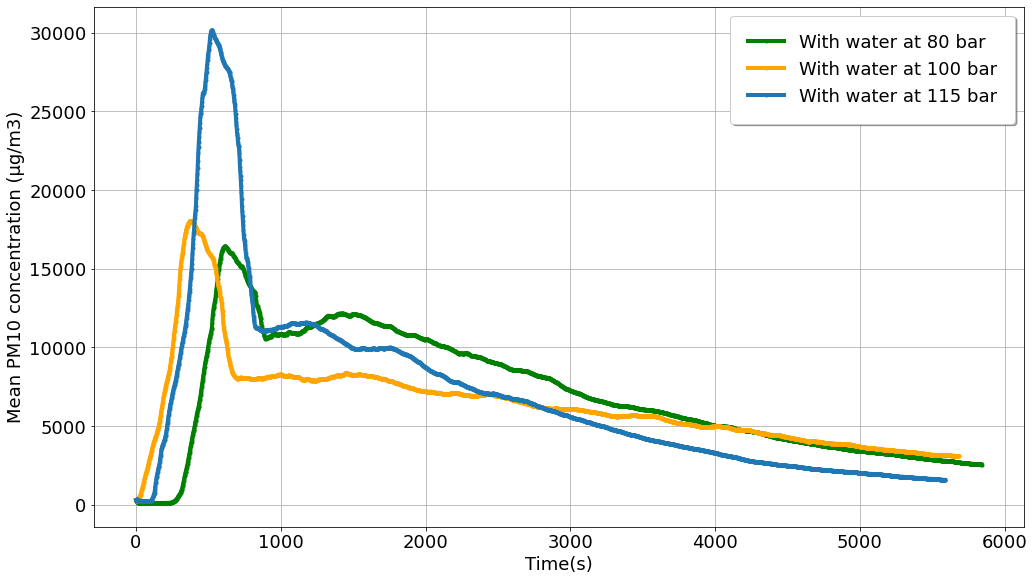


**S1.** Mean PM10 concentration for cutting activity with water (sprayed at 80 bar, 100 bar, and 115 bar pressures) as the control measure


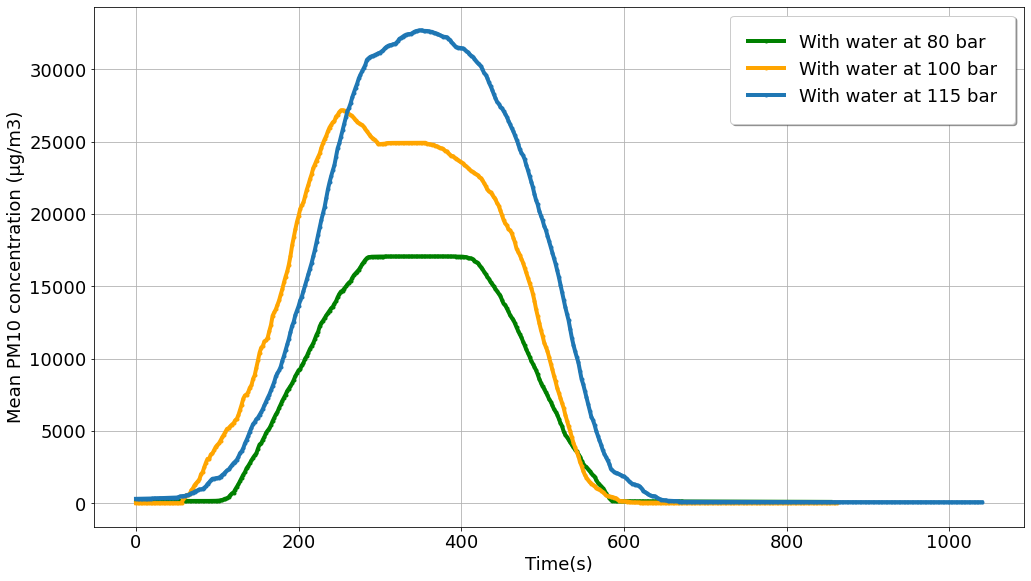


**S2.** Mean PM10 concentration from experiments with water sprayed at 80 bar, 100 bar, and 115 bar pressures

**S3**. Efficiencies of targeted PM control measures (water and dust suppressant spraying) compared to the case without any control measures

| **Percent. of PM reduction** | **Time required (s)** | **Time required (s)**  **(Efficiency - %)** | | | | | |
| --- | --- | --- | --- | --- | --- | --- | --- |
|  | **Without control measure** | **With water at 80 bar** | **With dust suppressant at 80 bar** | **With water at 100 bar** | **With dust suppressant at 100 bar** | **With water at 115 bar** | **With dust suppressant at 115 bar** |
| **30%** | 3048 | 608 (80.05) | 785  (74.25) | 324 (89.37) | 499  (83.63) | 461 (84.88) | 1095  (64.07) |
| **50%** | 4610 | 2494 (45.90) | 3389  (26.49) | 378 (91.80) | 868  (81.17) | 517 (88.79) | 2689  (41.67) |
| **70%** | 4939 | 3755 (23.97) | 5295  (0%) | 3373 (31.71) | 3264  (33.91) | 1669 (66.21) | 3946  (20.11) |
| **78%** | 6014 | 4524 (24.78) | - | 4371 (27.32) | 4366  (27.40) | 2327 (61.31) | 4666  (22.41) |


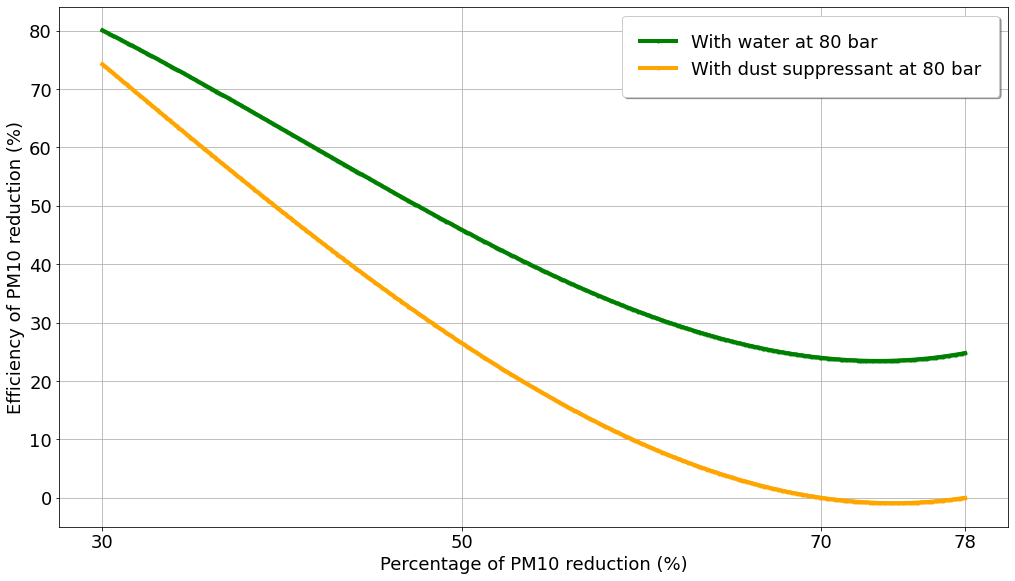


(a)


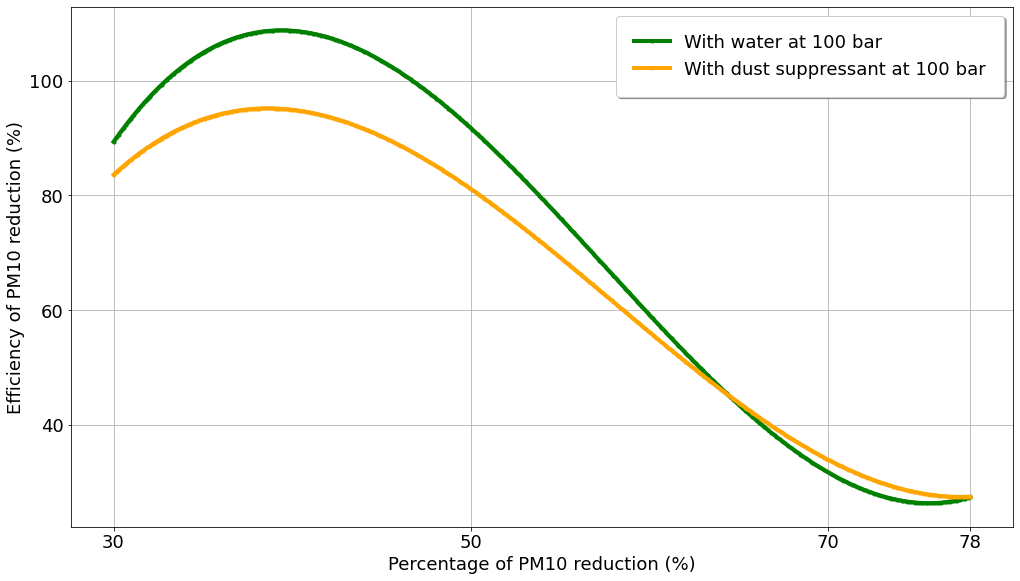


(b)


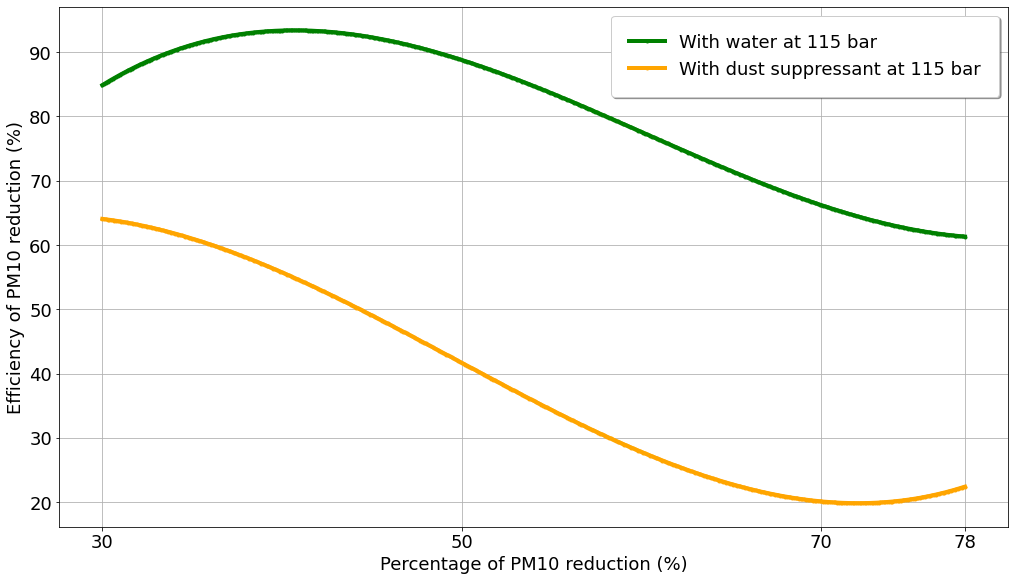


(c)

**S4.** Efficiency of PM10 reduction for water and dust suppressant sprayed at (a) 80 bar, (b) 100 bar, and (c) 115 bar pressures
